# Effect of Periodic Granulocyte Colony-Stimulating Factor Administration on Endothelial Progenitor Cells and Different Monocyte Subsets in Pediatric Patients with Muscular Dystrophies

**DOI:** 10.1155/2016/2650849

**Published:** 2015-12-06

**Authors:** Andrzej Eljaszewicz, Dorota Sienkiewicz, Kamil Grubczak, Bożena Okurowska-Zawada, Grażyna Paszko-Patej, Paula Miklasz, Paulina Singh, Urszula Radzikowska, Wojciech Kulak, Marcin Moniuszko

**Affiliations:** ^1^Department of Regenerative Medicine and Immune Regulation, Medical University of Bialystok, 15-269 Bialystok, Poland; ^2^Department of Pediatric Rehabilitation and Center of Early Support for Handicapped Children “Give a Chance”, Medical University of Bialystok, 15-274 Bialystok, Poland; ^3^Department of Immunology, Medical University of Bialystok, 15-269 Bialystok, Poland; ^4^Department of Allergology and Internal Medicine, Medical University of Bialystok, 15-276 Bialystok, Poland

## Abstract

Muscular dystrophies (MD) are heterogeneous group of diseases characterized by progressive muscle dysfunction. There is a large body of evidence indicating that angiogenesis is impaired in muscles of MD patients. Therefore, induction of dystrophic muscle revascularization should become a novel approach aimed at diminishing the extent of myocyte damage. Recently, we and others demonstrated that administration of granulocyte colony-stimulating factor (G-CSF) resulted in clinical improvement of patients with neuromuscular disorders. To date, however, the exact mechanisms underlying these beneficial effects of G-CSF have not been fully understood. Here we used flow cytometry to quantitate numbers of CD34+ cells, endothelial progenitor cells, and different monocyte subsets in peripheral blood of pediatric MD patients treated with repetitive courses of G-CSF administration. We showed that repetitive cycles of G-CSF administration induced efficient mobilization of above-mentioned cells including cells with proangiogenic potential. These findings contribute to better understanding the beneficial clinical effects of G-CSF in pediatric MD patients.

## 1. Introduction

Muscular dystrophies (MD) are a heterogeneous group of muscle diseases characterized by progressive muscle weakness and wasting [1, 2]. Despite promising gene-based therapeutic approaches being tested in MD, there is no cure available and thereby the need for developing novel therapies is still warranted [3–7]. There are at least two physiological mechanisms for tissue regeneration: (a) cell renewal, the replacement of damaged cells by newly generated cells delivered from resident stem cells; (b) cell proliferation, the self-repair of terminally differentiated well-functioning cells. Moreover, tissue regeneration requires angiogenesis for microvascular network restoration and to provide nutrient and oxygen delivery [7, 8]. It should be noted that progressive decline in muscle strength is caused in part by impaired blood flow in dystrophic muscles. There is a substantial body of evidence indicating that vascularity of muscles is significantly decreased in MD subjects [7, 9–11]. In addition, the process of angiogenesis is impaired in the course of MD. Therefore, induction of dystrophic muscle revascularization should contribute to diminishing the effect from functional ischemia and decrease myocyte damage. Accordingly, the proper therapy for skeletal muscle regeneration in MD needs to consider both revascularization of the tissue and myofiber regeneration. Therefore, use of biological therapies is an interesting approach in the treatment of muscular dystrophies [12].

To date, experimental therapies mainly focused on Vascular Endothelial Growth Factor- (VEGF-) related strategies. It is well established that VEGF function as a potent promotor of angiogenesis and promyogenic factor. In dystrophin deficient muscles VEGF was shown to promote myofiber regeneration and protect cells from apoptosis [13]. Moreover, VEGF leads to an increased blood vessels permeability, induction of endothelial progenitor cell (EPC) migration, and proliferation [14]. Thus, it is possible that, at least partially, VEGF-related beneficial effects could be attributed to an increase in EPC numbers. On the other hand, VEGF administration should be closely monitored due to carcinogenic properties [15, 16]. Thus, it is tempting to hypothesize that therapeutic strategies aimed at selective enhancement of EPC in muscular dystrophies could provide an attractive alternative for VEGF treatment.

Notably, there is a growing body of evidence that monocytes/macrophages are also important players in muscle regeneration. It should be noted that two distinct and functionally different subpopulations of macrophages are present in regenerating muscle tissue, namely, MI (classically activated) and MII (alternatively activated) macrophages. MI macrophages are referred to as proinflammatory cells and are involved in immune activation, phagocytosis, and muscle cell lysis. In contrast, MII macrophages are usually considered to exert anti-inflammatory properties as they have been shown to regulate inflammatory cell function and participate in vascularization process. This subpopulation is able to support muscle cell regeneration, by inducing satellite cell proliferation and tissue revascularization [17]. However, in the course of muscular dystrophy, myofiber degeneration leads to muscle invasion by both MI and MII macrophages. Similar to tissue macrophages, activated blood monocytes may display both anti-inflammatory and proinflammatory activities. Partially, these differential activities of monocytes are associated with their distinct phenotypes delineated by differential expression of CD14 and CD16. Thus, classical CD14++CD16− monocytes exert mostly phagocytic activities while intermediate CD14++CD16+ and nonclassical CD14+CD16++ monocytes play numerous immunomodulatory functions [18, 19]. It should be emphasized that biological properties of macrophages depend to a large extent on monocyte activation and maturation process that occurs at the periphery [20]. Thus the examination of distribution of peripheral blood monocyte subsets allows for assessing the pattern of monocyte-related immune responses. However, despite potential role different monocyte subsets could play in muscle regeneration, their dynamic changes in the course of MD and MD-targeted therapies were not yet examined.

Recently, the members of our group demonstrated that G-CSF administration brought beneficial clinical effects in pediatric patients with MD [21]. G-CSF is a member of colony stimulating factors that regulate the growth and differentiation of granulocytes and was shown to induce skeletal myocyte development and regeneration [22, 23]. It is used routinely in clinical practice for the treatment of neutropenia and in conditioning donors before stem cell transplantation [24, 25].

Here we wished to assess the effects of repeated cycles of G-CSF administration on mobilization of bone marrow derived stem/progenitor cells (most specifically endothelial progenitor cells) and different monocyte subsets in pediatric patients with MD. In parallel, we set out to analyze the effects of G-CSF administration on angiopoietins that similarly to EPC are involved in angiogenesis (e.g., via mobilization of EPCs) or a marker that is associated with changes of monocyte/macrophage phenotype, namely, soluble CD163 (sCD163).

## 2. Materials and Methods

### 2.1. Patients

A total of eleven muscular dystrophy patients were enrolled in this study. Detailed clinical characteristics of all patients are summarized in Table 1. Patients received their current standard treatment which was supplemented only by administration of filgrastim (Neupogen, Amgen) at the following doses: 5 *μ*g/kg of body weight/day for five consecutive days (course 1). Such treatment course was repeated after 1 month (course 2) and after 2 months (course 3).

### 2.2. Extracellular Staining and Flow Cytometry

Fresh EDTA-anticoagulated whole blood samples were stained with a panel (Table 2) of mouse anti-human monoclonal antibodies, according to stain-and-then-lyse-and-wash protocol. Briefly, 100 *μ*L (for monocytes) and 600 *μ*L (for EPCs) of whole blood were stained with monoclonal antibodies and incubated for 30 min at room temperature, in the dark. Thereafter, erythrocytes were lysed by adding 2 mL of FACS lysing solution (BD), followed by 15 min incubation in the dark. Cells were washed twice with cold PBS (phosphate-buffered saline) and fixed with CellFix (BD Biosciences). Fluorescence-minus-one (FMO) controls were used for setting compensation and to assure correct gating. Specimen acquisition was performed using FACSCalibur flow cytometer (BD Biosciences). Obtained data were analysed using FlowJo ver. 7.6.5 software (Tree Star).

### 2.3. Cytokine Assay

Angiopoietin-1, Angiopoietin-2, and sCD163 levels in EDTA-plasma samples from patients with MD were quantified by means of commercially available enzyme-linked immunosorbent assays (ELISA). To determine sCD163 plasma levels all samples were initially diluted 1000-fold with reagent diluent (1% BSA (Sigma-Aldrich) in PBS). Next, the specimens were assayed using sCD163 DuoSet ELISA kit (R&D Systems), according to the manufacturer's instruction. In order to determine Ang-1 and Ang-2 levels samples were directly assayed using Ang-1 DuoSet ELISA kit and Ang-2 DuoSet ELISA kit (both from R&D Systems). Finally, the protein levels in the specimens were calculated from a reference curve generated by using reference standards. The samples were analyzed with automated light absorbance reader (LEDETEC 96 system). Results were calculated by MicroWin 2000 software.

### 2.4. Statistical Analysis

Statistical analysis was carried out using GraphPad Prism 6 (GraphPad software). Wilcoxon test was used to compare changes in monocytes and EPCs numbers and plasma protein levels in single treatment course. Kruskal-Wallis test with post hoc Dunn's multiple comparison test was used to determine differences between all treatment courses. Spearman correlation coefficient was used to determine correlations between plasma protein levels and cell subsets. The differences were considered statistically significant at *p* < 0.05. The results are presented as median (interquartile range).

## 3. Results

**Figure 1:**
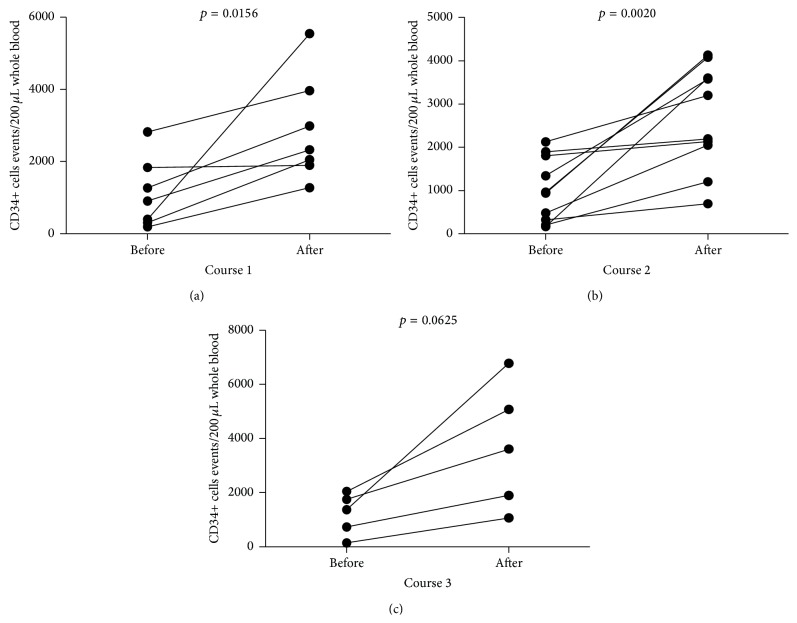
The summary of analyses of changes in CD34+ cells numbers after (a) course 1, (b) course 2, and (c) course 3 of G-CSF administration in MD pediatric patients.

First, we analyzed the effect of G-CSF treatment on hematopoietic stem/progenitor cells mobilization in children with MD. We observed substantial increase in CD34+ cell numbers after course 1 (from 908 (309–1839) to 2327 (1896–3965), Figure 1(a)), course 2 (from 951 (93.8–1827) to 2694 (1835–3720), Figure 1(b)), and course 3 (from 1368 (437–1895) to 3609 (1479–5930), Figure 1(c)) of G-CSF administration. Notably, repetitive courses of G-CSF treatment did not affect the efficiency of CD34+ cell mobilization in MD children (*p* > 0.05).

**Figure 2:**
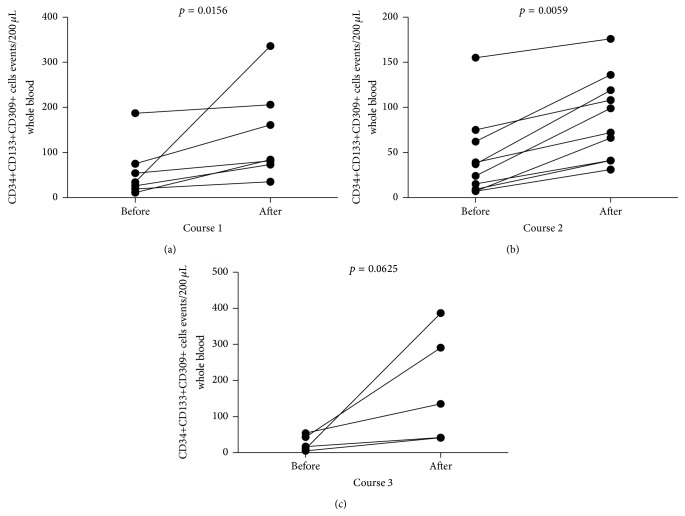
The summary of analyses of EPC numbers (expressing CD34+CD133+CD309+ phenotype) in MD pediatric individuals after (a) course 1, (b) course 2, and (c) course 3 of G-CSF administration.

**Figure 3:**
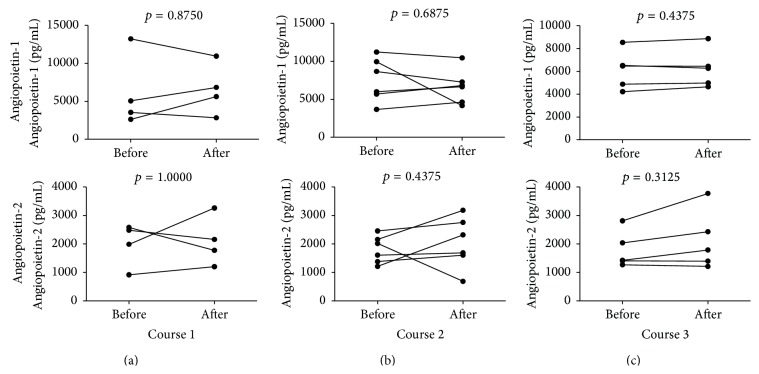
Time course changes in Ang-1 (upper row) and Ang-2 (bottom row) plasma levels in pediatric patients with MD after (a) course 1, (b) course 2, and (c) course 3 of G-CSF administration.

Next, we evaluated the numbers of endothelial progenitor cells (delineated by CD34+CD133+CD309+ phenotype) following repetitive courses of G-CSF treatment. We found significant increase in EPC numbers after course 1 (from 34 (19–75) to 84 (73–206), Figure 2(a)), course 2 (from 30.5 (8.5–65.2) to 85.5 (41–123.3), Figure 2(b)), and course 3 (from 17 (7.5–48.5)–135 (41.5–339), Figure 2(a)) of G-CSF administration. Again, no significant differences were observed in effectiveness of EPC mobilization between courses (*p* > 0.05). In parallel, we assessed the levels of two major angiopoietins, Ang-1 and Ang-2, during treatment with G-CSF and found that none of them was affected by this therapy (Figure 3).

**Figure 4:**
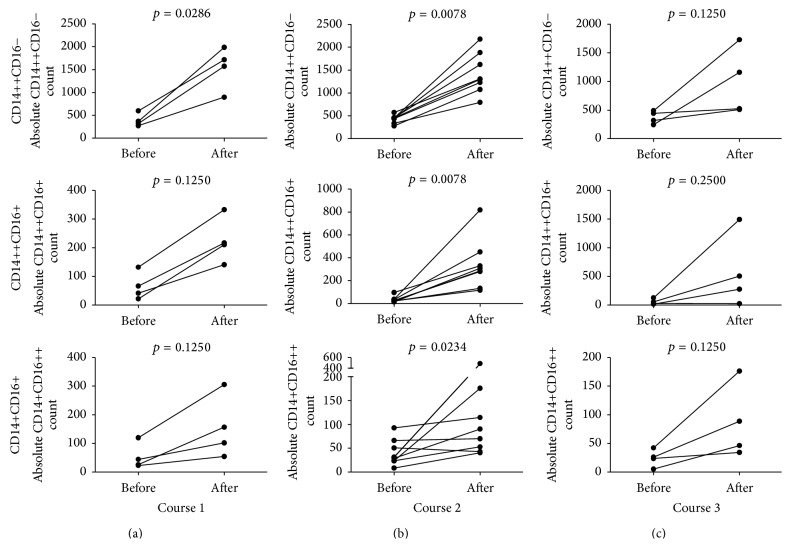
Effect of (a) course 1, (b) course 2, and (c) course 3 of G-CSF administration on absolute numbers of CD14++CD16− (upper row), CD14++CD16+ (middle row), and CD14+CD16++ (bottom row) monocytes in pediatric patients with MD.

Next we set out to investigate changes in absolute numbers of different monocyte subsets. We found that G-CSF administration induced mobilization of CD14++CD16−, CD14++CD16+, and CD14+CD16++ monocytes in all studied individuals (Figure 4(a)). Moreover, repeated administration of G-CSF also resulted in an increase in the numbers of all three above-mentioned subpopulations (Figures 4(b) and 4(c)). Interestingly, we did not observe any statistically significant changes of monocyte mobilization effectiveness between courses of treatment (*p* > 0.05).

**Figure 5:**
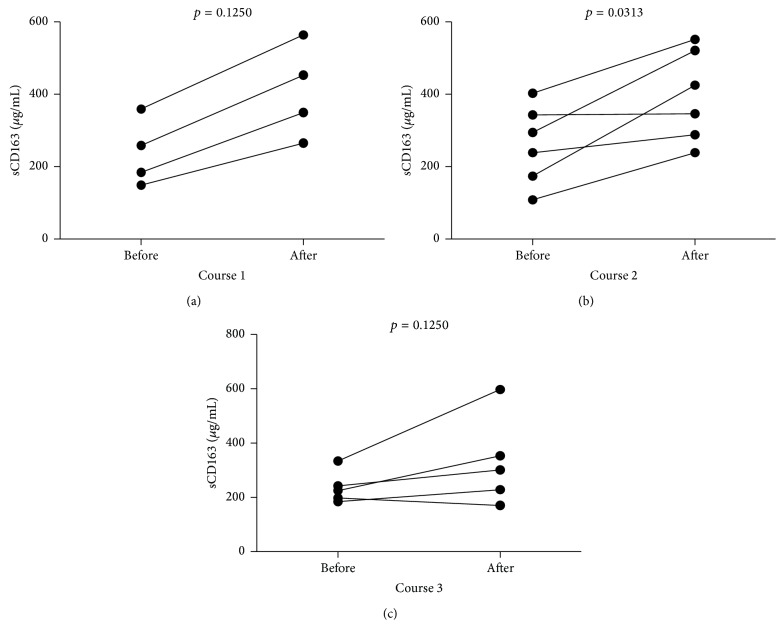
Time course changes in sCD163 plasma levels after (a) course 1, (b) course 2, and (c) course 3 of G-CSF administration in MD pediatric patients.

Next, we assessed the effects of G-CSF treatment on sCD163 levels. We observed substantial increase in sCD163 levels in all studied individuals undergoing initial treatment (Figure 5(a)). Interestingly, 5 out of 6 (83%) MD patients presented with an increase in sCD163 levels after course 2 (*p* < 0.05, Figure 5(b)). Moreover, 4 out of 5 (80%) MD children showed an increase in sCD163 levels after course 3 of GM-CSF administration (*p* > 0.05, Figure 5(c)). Again, there were no significant differences in effectiveness of treatment response based on sCD163 plasma levels (*p* > 0.05).

Finally, we investigated whether plasma Ang-1, Ang-2, and sCD163 levels were correlated to numbers of CD34+ cells, EPCs, and monocytes subsets in peripheral blood. We did not find any significant correlations among above-mentioned parameters.

## 4. Discussion

G-CSF-induced mobilization of hematopoietic stem/progenitor cells is usually delayed, with peak levels achieved within 5–7 days. In fact, in present study we observed a substantial increase of CD34+ cells, including hematopoietic stem cells (HSCs) (as expected, *p* = 0.015 for course 1; *p* = 0.007 for course 2; *p* = 0.031 for course 3; data not shown) and EPCs, in all studied individuals. Interestingly from clinical point of view, the growth rate of analyzed cell populations did not differ between courses of treatment (at monthly intervals). Similar to our study, de Kruijf et al. reported in mice model that multiple cycles of recombinant human G-CSF administration (up to 12 cycles) did not lead to bone marrow HSC pool depletion [26]. However, the long-term effects of repetitive or chronic G-CSF treatment on hematopoiesis and bone marrow steam/progenitor cells pool were not known. The contribution of CD34+ cells to muscle regeneration has been well documented [27–29]. However, CD34+ population is not uniform as it is composed of different subpopulations of progenitor/stem cells of which EPCs constitute a crucial subset involved in development of new vessels. Notably, we demonstrated here that G-CSF treatment of patients with pediatric MD increased EPC numbers in peripheral blood. This finding can be of importance in treatment of MD characterized by impaired vasculature. EPCs were shown to migrate in response to angiogenic growth factors, including angiopoietins to the site of ischemic tissue where they differentiate into mature endothelial cells (ECs). Thereafter, ECs proliferate to support and form new vessels. Furthermore, low dose CD34+VEGFR2+ cell transplantation hinders apoptotic cell death and reduces fibrosis in the ischemic muscles. These cells support ischemic muscle regeneration, improve the clinical outcome, and accelerate the hemodynamic recovery rate [28]. We showed here that repetitive use of G-CSF could contribute to improved “endothelization” of dystrophic muscles via efficient mobilization of EPCs. Given these promising data, further mechanistic studies defining in detail the role of EPCs in muscle regeneration in humans are still warranted. In addition, further studies in MD patients focused on measurements of possible triggering factors for progenitor cells such as stromal cell-derived factor-1 (SDF-1) or sphingosine-1-phosphate (S1P) would be of potential clinical benefit. Similarly, given the significant effects of G-CSF on progenitor cells, more detailed experiments addressing the effects of G-CSF administration on mobilization of stem cells subsets such as mesenchymal stem cells (MSCs) or very small embryonic-like stem cells (VSELs) in MD patients would be of significant interest.

We reported here substantial increase of all absolute monocyte subset numbers following G-CSF administration. Similarly, G-CSF was found to increase monocyte numbers in mice [30]. Moreover, Capoccia et al. showed that G-CSF-mobilized monocytes stimulated angiogenesis at sites of ischemia [31]. This study did not describe mechanism of monocyte-related angiogenesis; however, it can be hypothesized that this action was dependent on increased numbers of these monocyte subsets with proangiogenic potential, namely, those bearing high levels of Tie2, receptor for angiopoietins. These monocytes are referred to as Tie2 expressing monocytes (TEMs). Tie2 receptor is also present on HSCs and EPCs indicating that these cells constitute target populations for angiopoietin-mediated actions [32–34]. Angiopoietin-1 (Ang-1) and Angiopoietin-2 (Ang-2) are the best known and are characterized of the four, so far discovered, angiopoietins. Angiopoietin-1 is the principal activator of Tie2; additionally, it stimulates the migration of endothelial cells* in vitro* and promotes satellite cell self-renewal [35]. In contrast, Ang-2 is its natural inhibitor, blocking Ang-1-dependent phosphorylation of Tie2 receptor, which is reflected by destabilization of blood vessels and constitutes the initial stage of neovascularization [36, 37]. It should be noted that TEMs in the vast majority express CD16; therefore they fall into both intermediate and nonclassical monocytes [33, 38]. Here we found that G-CSF treatment increased both above-mentioned subpopulations; however, it did not affect Ang-1 and Ang-2 plasma levels. Thus we can hypothesize that G-CSF treatment increased monocyte numbers with proangiogenic potential in an angiopoietin-independent manner. However, further studies are warranted to explore whether such increase in both subpopulations of CD16-expressing monocytes could directly contribute to improved muscle regeneration in MD.

Quite surprisingly, we found here that G-CSF treatment tended to increase sCD163 levels. As surface CD163 can be shed from monocytes to become soluble CD163, one could hypothesize that enhanced levels of sCD163 following G-CSF therapy could result from enhanced levels of CD163 bearing monocytes (mostly classical and intermediate ones, see [18]). Interestingly, sCD163 has been considered as a surrogate marker of on-going monocyte-related inflammation [39]. Thus, it needs to be further examined whether G-CSF administration could be linked to enhancement of inflammation. However, on the other hand elevated sCD163 levels could have originated from alternatively activated macrophages (MII) known to have derived most frequently from intermediate monocytes. Previously, we have shown that intermediate monocytes expressed highest levels of CD163 [18]. Thus, G-CSF-induced enhancement of intermediate monocytes could have resulted in subsequent increase of MII macrophages known to exert beneficial effects on muscle regeneration. Nevertheless, potential use of sCD163 as a putative marker of enhanced muscle repair related to accumulation of MII macrophages needs to be clarified in further studies.

## 5. Conclusion

In summary, to our knowledge, this is the first report showing that repetitive G-CSF treatment can induce efficient mobilization of cells with proangiogenic potential, namely, EPC and putative proangiogenic monocytes. These findings could help better understand the beneficial clinical effects of repetitive G-CSF administration in MD pediatric patients. Nevertheless, the clinical safety of such treatment in this group of patients needs to be carefully addressed in further follow-up studies.

## Figures and Tables

**Table 1:** Clinical characteristics of studied patients.

Patient	Gender	Age (years)	Type of muscular dystrophy	Functional status
1	Boy	12	DMD	Nonwalking
2	Boy	11	DMD	Walking
3	Boy	12	DMD	Walking
4	Girl	13	FSHD	Walking
5	Boy	12	DMD	Nonwalking
6	Boy	15	BMD	Walking
7	Girl	10	MCMD	Nonwalking
8	Boy	11	DMD	Walking
9	Girl	15	FSHD	Walking
10	Boy	5	DMD	Walking
11	Boy	4	DMD	Walking

BMD: Becker musculardystrophy; DMD: Duchenne muscular dystrophy; FSHD: Facioscapulohumeral muscular dystrophy; MCMD: Merosin*-*negative congenital muscular dystrophy.

**Table 2:** Monoclonal antibodies used for flow cytometry analysis.

Specificity	Fluorochrome	Origin	Clone	Supplier
CD14	PE	Mouse	M*φ*P9	Becton Dickinson
CD16	FITC	Mouse	B73.1	Becton Dickinson
CD34	FITC	Mouse	581	Becton Dickinson
CD45	PE	Mouse	HI30	Becton Dickinson
CD133	APC	Mouse	AC133	Miltenyi Biotec
CD309	PE	Mouse	89106	Becton Dickinson
